# Structural Factors Affecting Health Examination Behavioral Intention

**DOI:** 10.3390/ijerph13040395

**Published:** 2016-04-01

**Authors:** Hui-Ting Huang, Yu-Ming Kuo, Shiang-Ru Wang, Chia-Fen Wang, Chung-Hung Tsai

**Affiliations:** 1 Taiwan Adventist Hospital, Taipei 401, Taiwan; gih@tahsda.org.tw (H.-T.H.); 116426@tahsda.org.tw (C.-F.W.); 2 Department of Marketing and Distribution, Tzu Chi University of Science and Technology, Hualien 970, Taiwan; tzustu@gmail.com; 3 Department of Business Administraiton, National Dong Hwa University, Hualien 974, Taiwan; shiang2216@gmail.com; 4 Department of Health Administration, Tzu Chi University of Science and Technology, Hualien 974, Taiwan

**Keywords:** health examination, health belief model, self-efficacy, social support, health knowledge

## Abstract

Disease screening instruments used for secondary prevention can facilitate early determination and treatment of pathogenic factors, effectively reducing disease incidence, mortality rates, and health complications. Therefore, people should be encouraged to receive health examinations for discovering potential pathogenic factors before symptoms occur. Here, we used the health belief model as a foundation and integrated social psychological factors and investigated the factors influencing health examination behavioral intention among the public in Taiwan. In total, 388 effective questionnaires were analyzed through structural model analysis. Consequently, this study yielded four crucial findings: (1) The established extended health belief model could effectively predict health examination behavioral intention; (2) Self-efficacy was the factor that most strongly influenced health examination behavioral intention, followed by health knowledge; (3) Self-efficacy substantially influenced perceived benefits and perceived barriers; (4) Health knowledge and social support indirectly influenced health examination behavioral intention. The preceding results can effectively increase the acceptance and use of health examination services among the public, thereby facilitating early diagnosis and treatment and ultimately reducing disease and mortality rates.

## 1. Introduction

According to a World Health Organization (WHO) survey, the ten top causes of death in the world between 2000 and 2002 were, in descending order, ischemic heart disease, chronic obstructive pulmonary disease, lower respiratory tract infection, tracheal or bronchial and lung cancer, AIDS, dysentery, diabetes, traffic accidents, and hypertensive heart disease [1]. According to statistics from Taiwan’s Ministry of Health and Welfare, the ten top causes of death in Taiwan in 2014 were, in descending order, cancer, heart disease, cerebrovascular disease, pneumonia, diabetes, accident injuries, chronic lower respiratory disease, hypertensive disease, chronic liver disease and cirrhosis, and nephritis, nephritic syndrome, and nephropathy [2]. Disease prevention and health promotion are critical public health concerns.

Advances in medical technology and social awareness have gradually increased health awareness among people. Disease screening instruments used for secondary prevention can facilitate early determination and treatment of pathogenic factors, effectively reducing disease incidence, mortality rates, and health complications. Therefore, people should be encouraged to undergo health examinations for detecting potential pathogenic factors before symptoms occur. Because health examinations can facilitate early diagnosis and corrective treatment, disease prevention should be the major focus, rather than treatment.

However, according to an investigation by the National Health Research Institutes (NHRI), although more than 70% of Taiwan residents aged older than 40 years know that the National Health Insurance Administration provides them free health examination services, 49.3% have never used them [3]. This indicates that the penetration of preventive health examination services in society requires improvement. Therefore, we constructed a research framework according to the health belief model in this study to investigate the factors that influence health examination behavioral intention among the adults. The study objective is to develop a research framework on the basis of the health belief model to investigate the factors influencing health examinations behavioral intention. This paper presents concrete strategies and recommendations for practitioners and future research.

### 1.1. Health Belief Model (HBM)

The health belief model, developed in 1950, is used to predict whether people can prevent and become aware of diseases, and it can facilitate resolving problematic behavior and prompting public health responses [4,5]). It assumes that people’s decisions to participate in health-related behavior are determined through six variables: (1) perceived susceptibility, which refers to the subjective assessment of the possibility of acquiring a certain disease or condition; (2) perceived severity, which refers to the assessment of the health impact of a disease; (3) perceived benefits, which indicate the assessment of the positive benefits of participating in health-promoting behavior; (4) perceived barriers, which refer to the beliefs on the difficulty and cost of participating in health-promoting behavior; (5) cues to action, which denote stimuli that promote action, can be external (such as advocacy through mass communication and advice from health-care workers and others) and internal (such as perceived physical discomfort and the appearance of disease symptoms); and (6) self-efficacy, which refers to people’s belief in their ability to engage in health-promoting behavior [6].

The preceding variables of the HBM, which can predict short- or long-term health-promoting behaviors, have been applied effectively to resolve various public health problems. For example, scholars have used them to explore young people’s exposure to tobacco smoke [7], osteoporosis prevention [8], healthy eating behaviors [6,9], diabetes self-care and diet [10], breast self-examination [11], and dieting behavior [12]. In addition, the model has been used to study health examination behavior during uterine cancer testing for effectively improving self-detection rates [13], mammography [14], human papillomavirus (HPV) vaccination [15], and dental care [16]. Thus, the HBM is applicable to various health behaviors.

However, the explanatory power of the HBM measurements is limited, and the model is insufficient for behavioral prediction, primarily because the average explanatory power (R^2^) of each variable is only approximately 20% [17]. In addition, they reviewed the relationship between specific HBM variables and behavioral outcomes [18] and concluded that the model’s prediction effectiveness is extremely low. Therefore, scholars have extended the variables of the HBM to strengthen its explanatory power; for example, studies on AIDS have included the variable HIV-related stigma in the model. However, most of the added variables are applicable only to specific research topics [6]. Therefore, we considered the factors that influence people who are engaged in a health examination behavioral intention in this study. Health knowledge and social support variables were added to comprehensively explore the factors influencing the public’s health examination behaviors and to extend the model’s application to various types of health examination.

### 1.2. Health Knowledge

Health knowledge refers to the individual’s storehouse of information about preventive health care behaviors [19]. It was proposed in a study on school education and health-promoting behaviors; that study indicated that schools and health are positively correlated, primarily because schools can improve knowledge of health-promoting behaviors and promote healthy living [20].

Health knowledge is positively correlated with health-promoting behaviors. For example, studies on AIDS prevention, health-promoting behaviors, hypertension prevention, and cancer screening have revealed that health knowledge substantially influences health-promoting behaviors [21,22,23,24]. Therefore, we included health knowledge in the research framework to investigate its influence on cues to action and behavioral intention.

### 1.3. Social Support

Social support refers to the perceptions of comfort, care, respect, or help from other people or groups [25]. Social support comprises: (1) self-esteem support: providing respectful messages of acceptance to other people, regardless of the difficulties they have encountered; (2) informational support: providing them with problem definitions as well as information and data required for understanding and processing problems; (3) social befriending: accompanying someone during spare time; and (4) instrumental support: offering financial assistance, equipment support, or necessary services [26]. Social support refers to positive social relationships that benefit a person who is encountering and struggling with challenges and stress that weaken self-esteem, causing a sense of powerlessness. When people are involved in positive social relationships, they can achieve psychological balance, which alleviates negative psychological states [27]. A study on the effect of social support and anxiety on health indicated that ample social support can effectively alleviate feelings of anxiety, improve health perceptions, and maintain healthy physiological and psychological states [28]. Therefore, we included social support in the current research framework to investigate its influence on cues to action and behavioral intention.

### 1.4. Behavioral Intention

According to the reasoned action theory, attitudes and subjective norms result in the formation of behavioral intention, thereby influencing behaviors. Behavioral intention is a necessary step in the behavior implementation process. In other words, behavioral intention refers to the action tendency to adopt a certain behavior [29]. Cues to action, perceived susceptibility, perceived severity, perceived benefits, perceived barriers, self-efficacy, health knowledge, and social support substantially influence behavioral intention [7,9,10,12,15,21,23,24,30,31,32,33,34,35,36]. Therefore, we used behavioral intention as the outcome variable to explore the influence of the variables of the HBM, health knowledge, and social support on health examination behavioral intention. The health examinations mentioned in this paper comprise physical examination and stool, urine, biochemical, and blood tests.

### 1.5. Relationships among Self-Efficacy, Perceived Benefits, and Perceived Barriers

Self-efficacy positively influences the frequency and intention of condom use. For example, although people know how to use condoms, they may feel apprehensive regarding their ability to use them. Therefore, in a study on the condom use behavior of female sex workers, those with higher self-efficacy showed more positive perceptions of condom use [36]. Therefore, Zhao *et al.* [36] hypothesized that self-efficacy and perceived barriers generate negative influences, whereas perceived benefits produce positive influences. The results of the present study were consistent with these hypotheses. Self-efficacy improves positive attitudes and perceptions of behaviors; thus, we propose the following hypotheses:

H1:  Self-efficacy positively influences perceived benefits.

H2:  Self-efficacy negatively influences perceived barriers.

### 1.6. Relationship between Social Support and Perceived Benefits

Chow and Chan [37] suggested that social participation provides increased opportunities for interpersonal contact. People reported positive feelings toward sharing ideas and resources with those with whom they had developed close relationships. When people engage in social activities, their social support typically increases correspondingly. For example, a study on heart diseases indicated that community health consultants and community residents frequently draw from the same social networks, life experiences, and cultural backgrounds to establish trust and maintain relationships that feature strong communication. Therefore, community health consultants use these links to share health information on blood pressure and health-care resources with the community, thus providing informational and instrumental behavioral support. This type of social support has positive effects, thus prompting community residents to improve blood pressure control and seek health care when required [38].

Chen *et al.* [30] and Tahmasbipour and Taheri [39] found mental health in young people have revealed that when young people perceive a high degree of social support, their mental health improves accordingly and stabilizes with their physical health. In addition, when people perceive strong social support, substantial positive influences on perceived happiness and health-related quality of life are observed [40,41]. Thus, perceived social support improves perceived health benefits; we propose the following hypothesis:

H3:  Social support positively influences perceived benefits.

### 1.7. Relationships among Health Knowledge, Social Support, and Cues to Action

Chen *et al.* [30] reported that people with lower education levels who live in remote areas possess a relatively poor understanding of the risks of hypertension and do not use salt-restriction spoons or engage in other health-promoting behaviors; moreover, people with higher education levels and higher family incomes who live in more urbanized areas are more accepting of and sensitive to cues to action. Therefore, a lack of health knowledge results in the potential neglect of diseases, hindering the reception of external information and adoption of effective preventive health-promoting behaviors. Marquez *et al.* [42] demonstrated that when social network resources are abundant, people are more likely to receive recommendations for recreational sports from friends and family than when these resources are scant. These results indicate that more health knowledge and social network information increase the accessibility to and acceptance of cues to action; thus, we propose the following hypotheses:

H4:  Health knowledge positively influences cues to action.

H5:  Social support positively influences cues to action.

### 1.8. Relationships among Perceived Severity, Perceived Susceptibility, Perceived Benefits, Perceived Barriers, Self-Efficacy, and Behavioral Intention

The HBM can be used to measure health-promoting behaviors. For example, Orji and Mandryk [9] reported that when people understand the potential perceived severity, perceived susceptibility, and perceived barriers of unhealthy eating behaviors, self-efficacy—the most influential factor—increases, resulting in increased healthy eating behaviors. A study on salt-restriction spoon use determined that the perceived severity of hypertension and perceived benefits of salt-restriction spoon use indirectly influence the use of these spoons, whereas perceived barriers directly influence salt-restriction spoon use. When perceived barriers increase, the use of salt-restriction spoons decreases substantially [30]. Furthermore, a study on iron-fortified soy sauce consumption indicated that when perceived susceptibility and perceived severity of iron deficiency anemia increase, consumer behavioral intention to consume iron-fortified soy sauce also increases [35]. In addition, a study on dieting revealed that self-efficacy substantially and positively influences dieting intention [12].

Regarding health-promoting behaviors, a study on the exposure of young people to environmental tobacco smoke suggested that when the perceived susceptibility of young people increases, they avoid environments with smoke to prevent passive smoking [7]. A study on condom use among female sex workers indicated that self-efficacy indirectly influences condom use: self-efficacy negatively and positively influences condom use through perceived barriers and perceived benefits, respectively [36]. Moreover, a study on car seatbelt use determined that increased perceived benefits substantially and positively influence seatbelt use. By contrast, when perceived barriers increase, seatbelt use decreases [34]. Furthermore, a study on helmet use revealed that when the perceived severity of potential traffic accidents increases and perceived barriers to wearing helmets decrease, young people are more likely to use helmets; however, perceived susceptibility and perceived benefits do not significantly influence behavioral intention [32]. Thus, the HBM can be applied effectively to various health-related research topics. Perceived susceptibility, perceived severity, perceived benefits, perceived barriers, and self-efficacy significantly influence behavioral intention; thus, we propose the following hypotheses:

H6:  Perceived severity positively influences behavioral intention.

H7:  Perceived susceptibility positive influences behavioral intention.

H8:  Perceived benefits positively influence behavioral intention.

H9:  Perceived barriers negatively influence behavioral intention.

H10:  Self-efficacy positively influences on behavioral intention.

### 1.9. Relationship between Cues to Action and Behavioral Intention

A study on iron-fortified soy sauce consumption behavior demonstrated that when potential consumers receive related cues to action, such as advertising information indicating that iron-fortified soy sauce can effectively promote health among all family members without causing negative effects, consumption behavioral intention increases [35]. Moreover, a study on helmet use indicated that when young people receive cues to action, such as when helmets are placed in conspicuous locations, their intention to use helmets increases substantially [32]. Therefore, cues to action influence behavioral intention; thus, we propose the following hypothesis:

H11:  Cues to action positively influence behavioral intention.

### 1.10. Relationship between Social Support and Behavioral Intention

Social support influences health-promoting behavior. For example, a study on unhealthy weight control determined that when young people lack social support from families and classmates, unhealthy weight control behaviors, such as fasting and avoiding breakfast, increase. The results indicated that establishing health knowledge and improving social support can encourage young people to healthily maintain their weight [31]. Nieminen *et al.* [33] revealed that when people have higher degrees of social support, health-promoting behaviors—including not smoking, engaging in recreational sports, eating fruits and vegetables, and sleeping adequately—increase substantially. Stanton and Campbell [28] reported that when people perceive higher degrees of social support and have more friends, healthy eating behaviors, such as the consumption of red meat, fruit, and vegetables, increase substantially, along with physical activity frequency. Gillibrand and Stevenson [10] revealed that family support facilitates improving self-care dieting behavior, enabling effective diabetes control. McKinley and Wright [43] determined that among university students, informational social support indirectly influences healthy dieting behaviors through site search frequency and site visits. Improving health awareness among students can increase health information searches, thereby facilitating healthy diet consumption, resulting in more healthy lives. The results of these studies have indicated that social support influences health-promoting behavior; thus, we propose the following hypothesis:

H12:  Social support positively influences behavioral intention.

**Figure 1:**
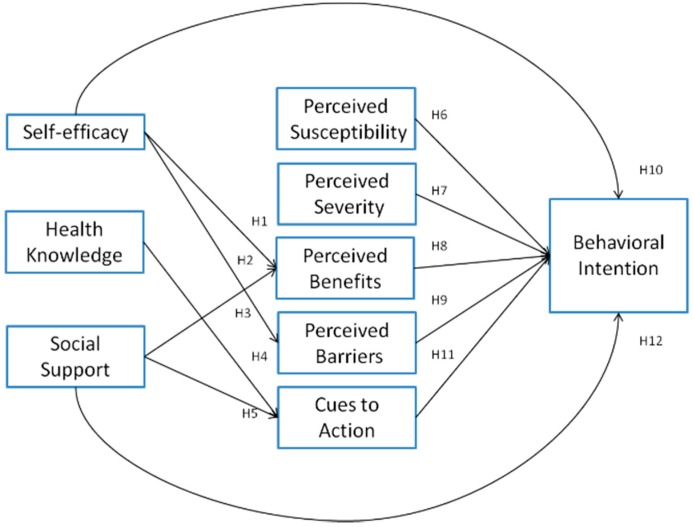
Research model.

All of the above assumptions shown in Figure 1.

## 2. Method

### 2.1. Questionnaire Design

The content of this study survey comprised 10 parts. The first part inquired about respondents’ basic information including sex, age, education, average monthly income, marital status, residence, number of children, and religion. The remaining parts were ordered as follows: (1) Self-Efficacy; (2) Health Knowledge; (3) Social Support; (4) Perceived Susceptibility; (5) Perceived Severity; (6) Perceived Benefits; (7) Perceived Barriers; (8) Cues to Action; and (9) Behavioral Intention. This entire survey used a 5-point Likert Scale for scorekeeping and quantification, with options of *Strongly Disagree*, *Disagree*, *Neutral*, *Agree*, and *Strongly Agree* assigned 1 to 5 points, respectively. In total, this study had 42 questions.

### 2.2. Participants

This study was conducted in Taipei and Hualien in Taiwan. Compared with other major Taiwanese cities, Taipei and Hualien have the highest and lowest population densities, respectively. We used the *t*-test for independent means to determine whether there is a statistically significant difference between Taipei and Hualien. The result showed that there is no significant difference Thus, the data from these cities constitute a sufficiently representative sample of Taiwan.

After Institutional Review Board approval (102-E-15) from both hospitals, and all participants signed an informed consent form, data were collected between October 2013 and March 2014. To prevent population liquidity and sporadic flu cases, the inclusion criteria were as follows: (1) aged 18–65 years; (2) residing for longer than 1 year in that city; and (3) not undergoing the first treatment at that particular hospital. We used a structured face-to-face interview survey with patients receiving health examination at three self-paid health checkup centers. The interviewers randomly visited participants, inquiring whether they wanted to participate in this study. If they agreed to participate, then they were asked to complete the questionnaire according to their health examination experience.

### 2.3. Research Instrument

In this study, the questionnaire was constructed using scales from previous studies. In other words, multiple items were used to measure each construct. We defined self-efficacy as a personal belief in the ability to engage in preventive health examination behaviors. Self-efficacy was measured using three items provided by Jayanti and Burns [19] (Cronbach’s α = 0.99). Moreover, we defined health knowledge as the information people possess on preventive health examination behaviors, which was also provided by Jayanti and Burns [19] (Cronbach’s α = 0.91). Furthermore, we defined social support as the comfort, care, and respect perceived by people or the assistance received from other people or groups during a health examination. This support included emotional and informational support, befriending, and instrumental support, all of which were provided by Griffin [44]. We adopted an integrated view to define perceived susceptibility, perceived severity, perceived benefits, perceived barriers, and cues to action. These measurement items were modified from those of McClenahan *et al.* [45]. The Cronbach’s α values of all the scales ranged from 0.75 to 0.85.

### 2.4. Internal Consistency, Convergent Validity and Discriminant Validity

Cronbach’s α coefficient was used to determine internal consistency. We also used the confirmatory factor analysis for testing convergent validity [46]. If the correlation coefficient of two dimensions is smaller than the square root of the average variance extracted (AVE) of each dimension, then it shows that each dimension has discriminant validity [47].

### 2.5. Statistical Analysis

We used structural equation modeling (SEM) to validate the model and our hypotheses. SEM is a statistical method involving a confirmatory approach for analyzing a structural theory bearing on a particular phenomenon [48]. Data analyses were performed according to the two-step approach recommended by Anderson and Gerbing [49]. First, the assessment of the measurement model comprising nine latent factors included the reliability, discriminant validity, and convergent validity of the scales. Second, the structural model was validated individually by considering the series of path relationships linking the nine constructs.

We followed the criteria provided by Weston and Gore [50] for examining whether the hypothesized model fit the observed data: (a) a root mean square error of approximation (RMSEA) of less than 0.05 indicates a good fit; (b) a comparative fit index (CFI) of more than 0.90 indicates a good fit; and (c) a chi-squared (χ^2^) fit index divided by degrees of freedom (χ^2^/df) of less than 3 on the normed χ^2^ test indicates a good fit [50]. If all the indices exhibit values close to or higher than the presented cutoff values, then the model is generally accepted to fit the observed data [51].

## 3. Results

### 3.1. Demographic Characteristics

As shown in Table 1, 157 (40.5%) of the participants were men, whereas 231 (59.5%) were women; in addition, 108 (27.8%) were aged 40–49 years—the largest age group—and 85 (21.9%) were aged 30–39 years—the second largest age group. Regarding education levels, those with college (junior college) degrees were the largest group, comprising 42.5% (165 people); and those with senior high school (vocational high school) degrees were the second largest group, comprising 24.2% (94 people). For income, most of the participants (27.3%) earned less than NT $25,000, followed by those (25.0%) earning NT $25,000–NT $44,999. Most of the participants (72.7%) were married, and 64.7% of the participants resided in Taipei, whereas 35.3% resided in Hualien.

### 3.2. Measurement Model Results

First, the reliability analysis of the dimension scales (Table 2) showed that all the Cronbach’s α values exceeded the acceptable level of 0.7 [52]. Second, we used the confirmatory factor analysis for testing convergent validity and used the composite reliability and AVE of the latent variables to measure the convergent validity of the latent and observed variables [46]. The composite reliabilities of all the dimensions of this study exceeded 0.7 (Table 2), indicating that each measured latent variable was internally consistent and thus that all the dimensions of this study had favorable convergent validity. Finally, the criterion for discriminant validity mentioned above was satisfied by all values in this study (see Table 3, such as 0.84 > 0.39), thus it showed that all dimensions in this study had good discriminant validity.

### 3.3. Structural Model Results

We used the AMOS software (IBM Software, Armonk, NY, USA) for structural model analysis. Here, the overall model fit was used to assess the goodness-of-fit of the model to the observable data: χ^2^/df was 2.226, with a goodness-of-fit index (GFI) of 0.861, adjusted GFI of 0.835, nonnormed fit index of 0.853, CFI of 0.913, incremental fit index of 0.913, and RMSEA of 0.056. Therefore, the findings showed that the goodness-of-fit of the overall theoretical model was favorable.

### 3.4. Hypothesis Testing

**Figure 2:**
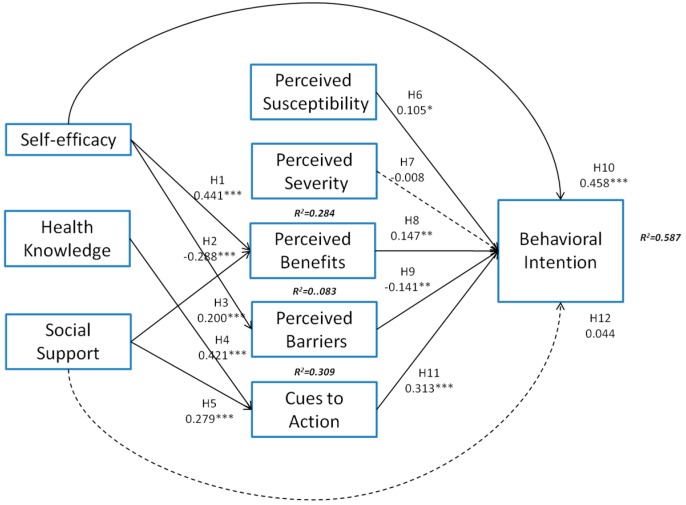
Final proposed model.

Most of the proposed hypotheses were supported by our results. Self-efficacy (path coefficient (β) = 0.441, *p* < 0.001) and social support (β = 0.200, *p* < 0.001) had a significantly positive influence on perceived benefits, accounting for 0.284% of the variance among the constructs; thus, H1 and H3 were supported. Self-efficacy (β = −0.288, *p* < 0.001) had a significantly negative effect on perceived barriers, accounting for 0.083% of the variance among the constructs; thus, H2 was supported. Health knowledge (β = 0.421, *p* < 0.001) and social support (β = 0.279, *p* < 0.001) had a significantly positive influence on cues to action, accounting for 0.309% of the variance among the constructs; thus, H4 and H5 were supported. Perceived susceptibility (β = 0.105, *p* < 0.05), perceived benefits (β = 0.147, *p* < 0.01), perceived barriers (β = −0.141, *p* < 0.01), self-efficacy (β = 0.458, *p* < 0.001), and cues to action (β = 0.313, *p* < 0.001) significantly affected behavioral intention, accounting for 0.458% of the variance among the constructs; thus, H6, H8, H9, H10, and H11 were supported. However, perceived severity and social support affected behavioral intention nonsignificantly, thus implying that H7 and H12 were not supported (Figure 2).

The structural model was also assessed through regression analyses. The results indicated that the significance of β between different factors and *R*^2^ were almost equivalent to that shown in Figure 2. Thus, although different statistical methods were used to test the hypotheses of this study, consistent results were obtained.

## 4. Discussion

According to our literature review, this is the first study to examine the relationship among self-efficacy, health knowledge, social support, perceived susceptibility, perceived severity, perceived benefits, perceived barriers, cues to action, and behavioral intention; we confirmed 10 of our 12 hypothesized relationships (Figure 2). The results of statistical analyses indicated that the factor exerting the greatest influence on health examination behavioral intention was self-efficacy, followed by social support and health knowledge.

The main value of the present study is that the explanatory power of 0.587 was achieved for health examination behavioral intention. The explanatory power of the measurements obtained using the HBM remains limited and insufficient for predicting behavior, primarily because the R2 values are 0.08–0.21 (approximately 20%), which is too low to predict the effects [17]. Although Carpenter [18] used meta-analysis to determine whether measures of these beliefs could consistently predict behavior again, the author suggested that future research can test some of the more complex versions of the model that may offer greater predictive power. Some scholars have continually extended the variables in the HBM to strengthen the model’s explanatory power. However, the majority of these added variables are applicable only to specific research topics [9]. In the present study, these variables could strongly predict influences but were not limited to specific research topics; thus, this model is broadly applicable to research related to various health examination items.

Our results indicated that active participation in health examinations increased when people trusted their ability to receive preventive health examinations, when their perceived social support increased, or when they possessed more health information. The results supported H1 and H2—according to the results of Zhao *et al.* [36]—confirming that self-efficacy substantially influences perceived benefits and perceived barriers.

Notably, the influence of perceived severity and social support on health examination behavioral intention was nonsignificant, most likely because we did not investigate patients with specific diseases. In other words, the respondents that did not have a disease that required urgent care did not perceive severity and thus lacked health examination behavioral intention. This corroborates the results of Orji and Mandryk [9] that some variables are applicable only to specific research topics.

### Implications

We recommend that health authorities and medical institutions strengthen health education pertaining to health examinations to increase public health knowledge. Straightforward methods can be used to facilitate the public understanding of the procedures involved in health examinations and the importance and effectiveness of disease prevention; thus, the related self-efficacy can be improved. In addition, hospitable and friendly environments can be created to enable people to perceive more social support and increase acceptance and use of health examination services. Moreover, we recommend that health screening centers produce videos to explain the processes, benefits, and costs of health examinations. Health examination advocacy should be conducted in businesses and within communities to increase people’s willingness to receive health examinations. Continual public care and warnings, along with channels and services for subsequent care, can enhance the sustainable health and welfare of people.

## 5. Conclusions

We used the HBM as the primary framework by adding the social and psychological factors of health knowledge and social support to investigate the factors influencing preventive health examination behavioral intention. Of the 12 hypotheses of this study, only the two regarding the influences of perceived severity and social support on behavioral intention were not supported. This indicates that the extended HBM constructed in this study can be used to effectively predict health examination behavior among people. Medical institutions and health authorities can refer to these results concerning health examination behavior to enhance public health and welfare. The future of national health can be improved by facilitating early diagnosis and treatment and reducing disease incidence and mortality.

## Figures and Tables

**Table 1:** Sample characteristics.

Item	N	%
Gender		
Male	157	40.5
Female	231	59.5
Age		
Less than 30 years old	53	13.7
30–39 years old	85	21.9
40–49 years old	108	27.8
49–50 years old	72	18.6
50–59 years old	43	11.1
over than 60 years old	27	7
Education		
Below junior high school	46	11.9
Senior high school (vocational high school)	165	42.5
College (junior college)	94	24.2
Above Master	83	21.4
Income		
less than NTS 25,000	106	27.3
between NTS 25,000 and 44,999	97	25.0
between NTS 45,000 and 64,999	80	20.6
between NTS 65,000 and 84,999	65	16.8
more than NTS 85,000	50	10.3
Marital status		
Married	282	72.7
Not Married	106	27.3
Place of residence		
Taipei	251	64.7
Hualien	137	35.3

**Table 2:** Internal consistency, convergent validity analyses.

Construct	Cronbach’s α	Composite Reliability	Average Variance Extracted
Self-efficacy	0.87	0.87	0.71
Health Knowledge	0.87	0.87	0.63
Social Support	0.89	0.89	0.54
Perceived Susceptibility	0.76	0.77	0.53
Perceived Severity	0.81	0.83	0.62
Percieved Benefits	0.89	0.89	0.68
Perceived Barriers	0.77	0.76	0.45
Cues to Action	0.80	0.80	0.49
Behavioral Intention	0.88	0.70	0.52

**Table 3:** Discriminant validity analyses.

Item	1	2	3	4	5	6	7	8	9
1. Self-efficacy	0.84								
2. Health Knowledge	0.39 ***	0.79							
3. Social Support	0.25 ***	0.20 ***	0.73						
4. Perceived Susceptibility	−0.03	−0.12 *	−0.05	0.73					
5. Perceived Severity	0.11 *	−0.13 **	0.24 ***	0.26 ***	0.79				
6. Percieved Benefits	0.42 ***	0.31 ***	0.28 ***	0.00	0.29 ***	0.83			
7. Perceived Barriers	−0.22 ***	−0.17 **	−0.06	0.23 ***	0.09	−0.10	0.67		
8. Cues to Action	0.40 ***	0.35 ***	0.28 ***	0.02	0.26 ***	0.60 ***	−0.07	0.70	
9. Behavioral Intention	0.58 ***	0.40 ***	0.24 ***	0.06	0.17 **	0.50 ***	−0.27 ***	0.53 ***	0.72

Sample size = 388; * *p* < 0.05; ** *p* < 0.01; *** *p* < 0.001.

## Appendix

### Self-Efficacy Scale

I usually attempt to eat a well-balanced diet.

I usually attempt to exercise regularly.

In the long term, people who care for themselves stay healthy.

People’s ill health results from their own carelessness.

In general, I can do things that make me healthy.

### Health Knowledge

Compared with an average person, I am more knowledgeable about taking care of my general health.

I am familiar with the techniques for preventing minor temporary problems, such as colds and viruses.

I am familiar with the techniques for preventing minor chronic problems, such as allergies and dry skin.

I am familiar with the techniques for preventing major temporary problems, such as flu and measles.

I am familiar with the techniques for preventing major chronic problems, such as hypertension and diabetes.

### Social Support

A special person cares for me when I am in need.

I can share my joy and sorrow with a special person.

My family really tries to help me.

I get the necessary emotional support from my family.

I have a special person who is the real source of comfort to me.

My friends really try to help me.

I can rely on my friends when things go wrong.

I can talk about my problems with my family.

I can share my joy and sorrow with my friends.

I have a special person who cares about my feelings.

I can talk about my problems with my friends.

My family is willing to help me make decisions.

### Susceptibility

My chances of becoming ill are greater if I do not perform self-examination.

Because of my physical health, I will more likely become ill if I do not perform self-examination.

I feel that my chances of becoming ill in the future are high if I do not perform self-examination.

I most likely will become ill if I do not perform self-examination.

I worry a lot about becoming ill if I do not perform self-examination.

### Severity

The thought of illness scares me.

When I think about illness, I feel nauseous.

If I had an illness, then my career would be endangered.

When I think about illnesses, my heart beats faster.

Illness would endanger my marriage (or a major relationship).

My feelings about myself would change if I become ill.

My financial security would be endangered if I become ill.

I am afraid to even think about illnesses.

I think I will experience an illness for a long time.

If I had an illness, my entire life would change.

### Benefits

I gain a lot benefits by performing self-examination.

Self-examination can help me find lumps related to my illness.

If I perform self-examination, then I may find a lump before it is discovered during a regular checkup.

### Barriers

Performing self-examination is embarrassing for me.

To perform self-examination, I must give up quite a bit comfortable.

Self-examination can be painful.

Self-examination can be time-consuming.

My family would mock me if I performed self-examination.

The practice of illness self-examination interferes with my activities.

Performing self-examination requires adopting a new habit, which is difficult.

I am afraid I would not be able to perform self-examination.

### Cues to Action

Doctor or nurse recommendations prompted me to perform self-examination.

Campaigns (e.g., media: press, TV, and radio) prompted me to perform self-examination.

Illness symptoms prompted me to perform self-examination.

Personal experience with illness prompted me to perform self-examination.

Family members or friends with illnesses prompted me to perform self-examination.

### Behavioral Intention

I intend to perform illness self-examination once a month.

I will attempt to perform illness self-examination in the next month.

I have decided to perform illness self-examination in the next month.
